# Supplementary material for: Effect of a hospital-wide campaign on COVID-19 vaccination uptake among healthcare workers in the context of raised concerns for life-threatening side effects
Source: PLoS One. 2021 Oct 1;16(10):e0258236. doi: 10.1371/journal.pone.0258236 (PMC8486118; doi:10.1371/journal.pone.0258236)
Supplement: S1 Appendix — (DOCX) [file pone.0258236.s001.docx]

**<<Questionnaire used in the study>>_Translated in English**

**Survey for healthcare workers who received first dose of vaccine**

**Baseline characteristics**

1. Age, years

2. Gender: female, male

3. Occupation: physicians, nurses, pharmacist, technician, others

4. Height, cm

5. Body weight, kg

6. Smoking: yes (including former smokers)/no

7. Alcohol: yes/no

8. Comorbidities

8.1 pulmonary disease: yes/no

8.2 renal disease: yes/no

8.3 neurologic disease: yes/no

8.4 diabetes mellitus: yes/no

8.5 cardiovascular disease: yes/no

8.6 malignancy: yes/no

8.7 autoimmune disease: yes/no

8.8 psychiatric disease: yes/no

8.9 allergic disease: yes/no

9. Family status

9.1 how many children do you have in your household?

none/one/two/more than three

9.2 do you live with their parents?

yes/no

**Adverse events after the first dose of ChAdOx1 nCoV-19 vaccine**

<Systemic reaction> (overwriting available)

1. Fever: yes/no

2. Vomiting: yes/no

3. Diarrhea: yes/no

4. Headache: yes/no

5. Fatigue: yes/no

6. Chill: yes/no

7. Myalgia: yes/no

8. Arthralgia: yes/no

9. Urticaria: yes/no

10. Dyspnea: yes/no

11. Shock: yes/no

<Local reaction> (overwriting available)

1. Injection site pain: yes/no

2. Injection site redness: yes/no

3. Injection site swelling: yes/no

<Damage caused by adverse events>

1. Did you experience decrease in work efficiency due to any adverse events?

yes/no

**COVID-19 experience and risk perception about COVID-19 severity**

1. Have you had been diagnosed with COVID-19?

yes/no

2. Have you had COVID-19 related symptoms (fever, cough, sore throat and so on) without confirmed diagnosis?

yes/no

3. Have you had tested for COVID-19 before?

yes/no

4. Do you know someone who had been diagnosed with COVID-19?

yes/no

5. How severe do you think you might get when you are infected with SARS-CoV-2?

mild/moderate/severe

**Perception about vaccines in the past**

1. Have you received the vaccines that experts recommend in the past? (For example, hepatitis A vaccine or tetanus vaccine if indicated)

A. always B. sometimes C. never

**Perception about COVID-19 vaccines**

1. Which vaccine do you think is most effective?

A. BNT162b2

B. mRNA-1273

C. ChAdOx1

D. Ad26.COV2.S

E. NVX-CoV2373

F. all the same

2. Which vaccine do you think is the most reliable?

A. BNT162b2

B. mRNA-1273

C. ChAdOx1

D. Ad26.COV2.S

E. NVX-CoV2373

F. all the same

3. Which vaccine do you want the most?

A. BNT162b2

B. mRNA-1273

C. ChAdOx1

D. Ad26.COV2.S

E. NVX-CoV2373

F. anything available

**Perception about ChAdOx1 nCoV-19 vaccine**

1. How much do you know about ChAdOx1 nCoV-19 vaccine?

A. had no interests

B. heard information from the media

C. looked for news and newsletters with interests

D. searched for literatures

2. Do you think that ChAdOx1 nCoV-19 vaccine is effective?

yes/no

3. Do you think that ChAdOx1 nCoV-19 vaccine is safe?

yes/no

4. Do you have concerns about the thrombotic thrombocytopenia syndrome associated with ChAdOx1 nCoV-19 vaccine?

yes/no

5. How often do you think the thrombotic thrombocytopenia syndrome associated with ChAdOx1 nCoV-19 vaccine occurs?

A. <1/1,000,000 B. 1/100,000~1/1,000,000 C. >1/100,000

6. Do you have confidence in the government's policy regarding vaccination (ChAdOx1 nCoV-19 vaccine is permitted for person over age of 30. However, when an individual had already received the first dose of the ChAdOx1 nCoV-19 vaccine, the person is allowed to get a second dose of the ChAdOx1 nCoV-19 vaccine regardless of their age.)?

turst/do not trust

7. Do you think that ChAdOx1 nCoV-19 vaccine is necessary?

8. Would you like to receive the second dose of ChAdOx1 nCoV-19 vaccine?

likely/undecided/unwilling to get vaccinated

**Survey for healthcare workers who completed second dose of vaccine**

1. When did you decide to receive the second dose?

A. before hospital-wide campaign

B. after hospital-wide campaign

2. What is main reason for decision?

A. resolved or relieved anxiety about second dose owing to hospital-wide campaign

B. national policy

C. recommendation by persons around

D. positive information from media

E. other

**<<Questionnaire used in the study>>_In original language**

**1차 접종 완료자를 위한 설문조사**

**기본 정보 조사**

1. 나이: 직접 기입

2. 성별: 남, 여

2.1 여성일 경우 백신 2차 접종 즈음에 임신 계획 여부: 예/아니오

3. 직종: 간호사, (치)의사, 약사, 방사선사, 병리사, 이송요원, 청소요원, 행정직, 기타

4. 키: 직접 입력

5. 몸무게: 직접 입력

6. 흡연: 예/아니오

7. 음주: 예/아니오

8. 과거력

8.1 호흡기 질환: 예/아니오

8.2 신장 질환: 예/아니오

8.3 신경계 질환: 예/아니오

8.4 당뇨: 예/아니오

8.5 심혈관계 질환: 예/아니오

8.6 암 질환: 예/아니오

8.7 자가면역 (류마트스질환): 예/아니오

8.8 정신과적 질환: 예/아니오

8.9 알레르기 과거력: 예/아니오

8.9-1 약제에 대해 알레르기가 있다면 다음 중 어떠한 것에 있는지 확인

A. 백신 B 약제 C 기타

8.9-2 알레르기의 정도는?

A경증 B 중등증 (진료가 필요한 정도) C 중증 (입원이 필요한 정도)

9. 가족관계

9.1 나는 아이가

A 없다. B 1명 C 2명 D 3명 이상

9.2 나는 같이 살고 있는 부모님이

A 있다. B 없다.

**백신 1차 접종 후 이상반응 조사**

<전신 반응> (중복 체크 가능)

1. 발열 : 예/아니오

2. 구토: 예/아니오

3. 설사: 예/아니오

4. 두통: 예/아니오

5. 피로감: 예/아니오

6. 오한: 예/아니오

7. 근육통: 예/아니오

8. 관절통: 예/아니오

9. 전신 두드러기: 예/아니오

10. 호흡 곤란: 예/아니오

11. 쇼크: 예/아니오

12. 위의 전신 이상반응으로 외래/ER 진료를 보았습니까? 예/아니오

13. 위의 전신 이상반응으로 입원을 했었습니까? 예/아니오

<국소 반응> (중복 체크 가능)

1. 주사 부위 통증: 예/아니오

2. 주사 부위 발적: 예/아니오

3. 주사 부위 부종: 예/아니오

4. 위의 국소 이상반응으로 외래/ER 진료를 보았습니까? 예/아니오

5. 위의 국소 이상반응으로 입원을 했었습니까? 예/아니오

<이상반응 관련 업무 피해 정도>

1. 백신 접종으로 인한 피해가 있었습니까? 예/ 아니오

① 정도: 업무 능률 25% 미만 감소/25-50% 감소/50% 이상/75% 이상 감소

**일반 백신에 대한 인식도 조사**

1. 나에게 추천되는 백신들(예를 들어, 더러운 상처에 파상풍 백신 혹은 해외 여행 전 필요한 백신; 단, 원내 독감 접종은 특수한 사항으로 고려하지 않음) 접종 권고에 대해

A 항상 잘 따랐다. B 가끔 따랐다. C 전혀 따르지 않았다.

**코로나 질병 관련 조사**

1. 나는 코로나-19로 진단받은 적이 있다: 예/아니오

2. 코로나19가 발표된 이래 확진 받은 적은 없지만 코로나19 의심 증상(발열, 호흡기 증상)을 겪어본 적이 있다: 예/아니오

3. 코로나19 의심 증상으로 코로나 검사를 받아본 적이 있다: 예/아니오

4. 주변에 코로나19로 진단받은 사람이

A 있으며 입원까지 했다. B 있지만 입원은 하지 않았다. C 없다.

5. 내가 코로나19는 걸리게 되면

A 매우 심각할 것이다. B 다소 심각할 수 있다. C 별로 심각하지 않을 것이다. D 경증일 것이다.

**코로나 백신 인식도 조사**

1. 어떤 백신이 가장 효과가 좋다고 생각하는가?

A 화이자 B 모더나 C 아스트라제네카 D 얀센 E 노바백스 F 다 똑같다

2. 어떤 백신이 가장 안전하다고 생각하는가?

A 화이자 B 모더나 C 아스트라제네카 D 얀센 E 노바백스 F 다 똑같다

3. 본인이 가장 희망하는 백신은?

A 화이자 B 모더나 C 아스트라제네카 D 얀센 E 노바백스 F 어느 백신이든 상관없다.

**아스트라제네카 백신 인식도 조사**

1. 아스트라제네카 백신에 대해서 얼마나 알고 있습니까?

A 잘 모른다 B 미디어에서 들은 정도로 알고 있다 C 관심을 갖고 직접 뉴스와 소식지를 찾아 본다 D 전문 문헌을 검색해 본다

2. 아스트라제네카 백신은 COVID-19을 예방하는 데

A 효과 있다 B 효과 없다

3. 아스트라제네카 백신 접종은

A 안전하다 B 안전하지 않다

4. 아스트라제네카 백신의 혈전 생성 관련 이상반응에 대해 걱정되는가? 예/아니오

5. 혈전 생성 관련 이상반응은 어느 정도의 빈도로 나타나는 것으로 알고 있는가?

A 1/1,000,000 미만 B 1/100,000~1/1,000,000 C 1/100,000 이상

6. 혈전 생성 관련 이슈로 변경된 정부의 백신 접종 정책(30세 이상에서만 아스트라제네카 백신 접종. 단, 이미 1차를 맞은 경우 30세 미만도 아스트라제네카 백신으로 접종 가능)에 대해서 어떻게 생각하는가?

A 신뢰한다 B 신뢰하지 않는다

7. 아스트라제네카 백신 2차 접종이 필요하다고 생각하는가?

A 그렇다 B 잘 모르겠다 C 그렇지 않다

8. 2차 백신을 접종할 것인가?

A 맞을 것이다. B 결정 못했다. C 맞지 않을 것이다.

**2차 백신 완료자를 위한 설문조사**

1. 2차 접종을 하기로 결정한 시기는?

A. 교직원 대상 캠페인 이전 (5월 18일 이후)

B. 교직원 대상 캠페인 이후 (5월 18일 이전)

2. 해당 시기에 2차 접종을 하기로 결정하게 된 가장 큰 원인은?

A. 교직원을 위한 이상반응 전문 진료 형성 및 원내 교육으로 막연한 불안감이 다소 해결

B. (언론이나 유튜브 등을 통해) AZ 백신에 대한 긍정적 정보 획득

C. 주변의 권유

D. 백신 인센티브 제공 때문에

E. 기타
