# Supplementary material for: Effect of a hospital-wide campaign on COVID-19 vaccination uptake among healthcare workers in the context of raised concerns for life-threatening side effects
Source: PLoS One. 2021 Oct 1;16(10):e0258236. doi: 10.1371/journal.pone.0258236 (PMC8486118; doi:10.1371/journal.pone.0258236)
Supplement: Attachment — Submitted filename: Review Report PONE-D-21-22598.pdf [file pone.0258236.s003.pdf]

**Review Report**

**Manuscript Number:** PONE-D-21-22598

**Title: Effect of a hospital-wide campaign on the COVID-19 vaccination uptake among healthcare workers**

**Background:**

This study focusses on the vaccine hesitancy of hospital workers in Republic of Korean context. It shows that hospital-wide campaign to motivate health workers to get vaccinated – lowered vaccine hesitancy among health workers in getting the second dose.

**Comments:**

While I enjoyed reading the paper, I have some major reservations.

First, I am not sure about the novelty of the study. It is well known that 'directed campaign' and 'generating awareness on vaccination', encourage people to get vaccinated. Such campaign is also seen as an important means to mitigate the adverse affect of vaccine misinformation. Therefore, while I acknowledge that this might be the first study on hospital workers on vaccine hesitancy, the results are not surprising. I would suggest the authors to focus more on the contribution of the study/ paper. In other words, what is the incremental leaning from this study? In this context, I would like to highlight that it should be easier to convince hospital workers (compared to general public) about the benefits of vaccination and lower their vaccine hesitancy.

Second, I have concerns about the survey design. As mentioned in the study, 1,171 (60.3%) of the total hospital workers (1,851) received vaccination during the period of 8 to 19 March, 2021. The survey (of this study) focusses on 1,171 health workers who received the first dose of vaccination. To me, it is more important to focus on the health workers who did not want to get vaccinated

(even the first dose). Because, those who got the first dose, know that without the second dose their vaccine will not be effective. Therefore, it is easier to manage the vaccine hesitancy challenge of this group. But the real challenge lies with the unvaccinated health workers (36.7% of total health workers!). I would suggest the authors to focus on this group as well – and explore what is the cause of vaccine hesitancy for this group (who did not even want to get the first dose).

Third, based on the univariate and multivariate analysis, the study has identified a few factors that contribute to vaccine hesitancy (female workers, younger workers…). However, no compelling arguments are presented by the authors as to why these factors lead to a higher level of vaccine hesitancy. As the general management literature suggests, female workers are more rule abiding, more compassionate and care more about society. It is not clear why female workers would be more hesitant. Similarly, it is not clear why younger workers would be more hesitant (may be, they have lower covid risk?). These points are important – because the hospital wide campaign should be linked to such factors.

Fourth, (somewhat linked to my previous point), there is no link discussed between the hospital wide campaign and the factors contributing to vaccine hesitancy. It looks like that any general campaign could work. This view questions the relevance of this study.
