# Supplementary material for: Effect of a hospital-wide campaign on COVID-19 vaccination uptake among healthcare workers in the context of raised concerns for life-threatening side effects
Source: PLoS One. 2021 Oct 1;16(10):e0258236. doi: 10.1371/journal.pone.0258236 (PMC8486118; doi:10.1371/journal.pone.0258236)
Supplement: Attachment — Submitted filename: Response to Reviewers_2 (Revision of PONE-D-21-22598)_0913.docx [file pone.0258236.s004.docx]

To the reviewers

Thank you for your detailed evaluation of our article. We have corrected the text based on your recommendations. Corrections are shown in red and our responses to your comments are shown below.

**Reviewer #2:**

*The authors report on a study on hesitancy to get vaccinated with the AstraZeneca vaccine among healthcare workers (HCW) in Yonin Severence Hospital in South Korea. HCW participated in online surveys after the first vaccine dose and were interviewed about vaccine hesitancy to receive a second doses. After the survey, a hospital-wide information campaign was launched to promote compliance with the second dose. After the second dose a second online survey was conducted among HCW to investigate the reasons for compliance with the second dose.

The topic is definitely relevant in the context of vaccination hesitancy, particularly in the context of the ongoing discussion of mandatory vaccines. Naturally, such studies have their limitations. The manuscript is well written and easy to understand. I think it has merit and should be published. However, I have a number of comments and suggestions that should definitely be addressed. I believe my comments can be easily addressed.*

*General comments:*

1. *Reasons to get vaccinated change rapidly throughout the pandemic. While some people did not want to get vaccinated immediately, their concerns naturally diminished over time, as more people were already vaccinated, aggressive variants are spreading, and incentives to get vaccinated are being implemented. This should be discussed a bit more.*

**Response:** Thank you for your detailed comment.

1. We agree to your opinion that concerns about vaccination would diminish naturally over time. Some of HCWs decided to get vaccinated with second dose regardless of our campaign, as shown in results of this study. However, our study highlights the effects of our campaign on responders who decided to receive a second dose after the hospital-wide campaign. Indeed, 57.1% of those responded that the hospital campaign was a major determinant in their decision to accept the second dose of vaccination.

2. We agree with your opinion regarding that vaccine incentives would have influenced on improving vaccine coverage. This has already been described in discussion section, on page 19, line 241.

3. Emergence of variants would likely to have influenced to get vaccinated with ChAdOx1 nCoV-19 due to reduced vaccine efficacy compared with mRNA vaccines manufactured by Pfizer or Moderna. However, it is difficult that variants could have influenced on vaccination with second dose in our study since variants were rarely observed in South Korea at the time this survey was conducted.

2. *Throughout the ms, the vaccine is called ChAdOx1 nCoV-19. It should be mentioned that it Is called also AZD 1222 or the AstraZeneca vaccine. I believe this will improve accessibility to a broader audience.*

**Response:** We agree with your comment, so we added the official name of the vaccine, which is Oxford-AstraZeneca adenovirus-vectored vaccine, at its first appearance.

In the Introduction section on page 3, line 71:

“The Oxford-AstraZeneca adenovirus-vectored vaccine (ChAdOx1 nCoV-19)”

3. *It should be discussed that the campaign can presumably not be scaled up to the full extend. It might work well in hospitals, but running such information campaigns in the general population is different, particularly point (4). However, some parts of the campaign are scalable. In so far this study gives insights in what could be done in the general population to promote the vaccine. I think the ms would profit from a broader discussion.*

   **Response:** We agree with your comment, so, we supplemented our article by adding discussion for the implication for general population. We revised discussion accordingly.

In the discussion section page 19, line 220-244:

“The second survey revealed that 76.7% of participants had decided to accept the second dose of the vaccine before the campaign began. Previous reports on HCWs in France also suggested a similar percentage of vaccine acceptance. In contrast to the general population [1], stronger intentions to receive vaccination have already been established among HCWs regardless of their job type. Responsibility as HCWs and high risk of exposure to COVID-19 might contribute to a favourable attitude towards the vaccine. This reflects the social consensus formed among HCWs confronting an unprecedented pandemic. Therefore, the HCWs of this study might be easily convinced of the necessity of vaccination and lower vaccine hesitancy.

Authentic information and dedicating resources to managing potential adverse events are important motivators for vaccination. Previous reports have revealed that misinformation often leads to vaccine hesitancy, which prompts the need for education of the public. The importance of education targeting HCWs has been emphasized in studies of other vaccines. We strived to correct misinformation related to ChAdOx1 nCov-19 vaccine through education. Several opportunities were provided to HCWs to be educated during a hospital-wide campaign. Such campaigns should be tailored to meet the needs of the public to address the ongoing challenge: For example, cases of TTS were reported among persons who had received the ChAdOx1 nCov-19 vaccine, after which many HCWs who had been vaccinated with first dose expressed concerns about vaccination with the second dose. Therefore, our campaign focused on addressing concerns related to TTS. In addition to providing accurate information about TTS, we developed a specialized clinical team so that HCWs can receive medical consultation promptly whenever they complained of any symptoms after the second dose. Most responders said that the hospital campaign was a major determinant in their decision to accept the second dose of vaccination. Although it was not determined which component of the campaign had the greatest effects on resolving the hesitancy for the second dose, we believe that education and a specialized clinical team for HCWs played a major role in addressing vaccine hesitancy. Our findings provide insight for the direction of national policies to improve vaccine coverage in the general population, especially among young adults who agonize over vaccination due to an imbalance in the associated risks and benefits of vaccination [2]. Further study evaluating the effects of tailored campaign on vaccine uptake in the general population is warranted.”

*Major comments:*

1. *I have major concerns regarding the statistical analyses. Looking at the numbers in Table 1, I did not believe the p-vales reported. Hence, I ran some tests in R and obtained totally different p-values (which seem plausible to me). For instance, smoking: I ran the following R-code:
   Fishers exact test:
   fisher.test(matrix(c(79,540,14,204),ncol=2,nrow=2))
   and obtained a p-value of 0.01171
   Chi-square test with Yates correction:
   chisq.test(matrix(c(79,540,14,204),ncol=2,nrow=2))
   p-value: 0.01483
   Chi-square test without Yates correction:
   chisq.test(matrix(c(79,540,14,204),ncol=2,nrow=2),correct = FALSE)
   p-value: 0.01042
   The reported p-value was <0.001

   It is similar for all other variables I checked. The p-values reported just do not seem plausible to me, and I obtained different p-values for all variables I checked.

   I believe it is necessary to revise the table and the main text accordingly.*

**Response:** Thank you for your comment. To make sure we had analyzed the data correctly, we double checked the result about smoking status using SAS. We reproduced p-value 0.0104, which was exactly the same figure as you had calculated with Chi-square test without Yates using program R. The results were also the same with the other two methods, Chi-square with Yates correction and Fishers exact test. We presented p-value by rounding it up to the decimal point 0.01. The result makes no difference whether you use Yates correction or not. I am afraid that you confused the number with that of the above. We present our results below.

Capture of the Table1 ‘Smoking’

2. *Table 1: Please be more specific on the statistical test. In all 2x2 tables a fisher’s exact should be performed. If a chi-square test is performed, it is unclear whether the Yates correction was applied (with this sample size I would not do it as it tends to over-correct).*

**Response:** According to a study by Ronald Fisher (1922), a Chi-squared test (or better yet, a G-test) can be used when the sample size is big. However, it only provides approximation because the distribution of the samples that is calculated is an approximation which is equal to the theoretical Chi-squared distribution. The approximation is invalid when the sample sizes are small or data are unequally distributed across the table, resulting in low counts on the cells predicated on a null hypothesis (the expected counts). The usual rule of thumb is that **chi-squared test is not suitable when the expected values in any of the cells of a contingency table are below 5, or below 10** when there is only one degree of freedom [3]. In our study, we annotated with lower case ‘a’ when Fisher’s exact test is required. We present the results below.

Capture of the Table 1 ‘Others’ and annotation

3. *Page 12: It needs to be specified more clearly what models were run. I suppose the univariate analyses means a logistic regression just with an intercept and this variable. With the multivariate analysis on the other hand all variables were forced into the logistic model.
   I have objections against both. By the univariate analyses, a lot of models are fit, all of them presumably with a poor fit. However, since there is just one covariate and the intercept, the estimated regression coefficient will always appear significantly different form 0. This is actually seen by the small p-values. By the multivariate analysis, the model tends to overfit. After correction for multiple testing hardly any odd ratio is significantly different from 1.
   An appropriate approach is to do a model selection based on the AIC criterium (in R function stepAIC – you specify the minimal model which is just and the maximum model). It will return the optimal model based on the AIC. The p-values of the z-tests for the regression coefficients that remain in the model should then be corrected for multiple testing by the Holm-method (I do not recommend the Hochberg method in this case).
   In any case the model fit needs to be reported in terms of AIC and Null-Deviance and Deviance. The logistic regression is only justified if the model properly fits.*

**Response:** We appreciate your comment. Here are our answers. First of all, the purpose of the second analysis was to figure out the factors that leads to hesitancy. We carried out logistic regression analysis as described in “Statistical analysis” section, confirming the significance of each variable with univariate analysis. With the variables of p-value 0.05 or below by global test as well as the ones with clinical relevance, we performed multivariate analysis. Validity of the variables was confirmed by statistical variable selection method. Generally, researchers use Akaike Information Criterion (AIC) or Bayesian Information Criterion (BIC) to find the fittest model among various ones the methods present. Furthermore, AIC and BIC is useful to prevent reckless addition of variables for the purpose of gaining fitness. In this sense, we assume using them to check the adjusted effect of the variables is inappropriate.

4. *Introduction/Methods (L90-97): other reasons for vaccine hesitancy could have been the fact that the former “South African” variant spread and the AstraZeneca is not properly protecting from that variant. Another reason for hesitancy for the second dose might have been, that at this time it was reported that a longer vaccination schedule would improve the vaccine’s effectiveness. This should be discussed.*

**Response:** Thank you for your comments. It is true that the efficacy of the ChAdOx1 nCov-19 vaccine against variants is lower than that of mRNA vaccine. However, at the time this survey was conducted, variants were rare in South Korea and were observed in only a few foreign entrants from overseas. Therefore, I think that the emergence of variants is less likely to influence hesitancy for second dose. Also, since the schedule of second dose was set to 11-12 weeks after the first vaccine (recommended longest time to be extended) in our institution, it is difficult for HCWs to hesitant to second dose to improve the efficacy of vaccine.

*Minor comments:*

1. *L81: vaccine coverage rate: what is the vaccine coverage rate? Is it really a rate (with unit 1/time) or is it the percentage of vaccinated individuals in a certain group?*

**Response:** Thank you for your detailed comment. We decided to use ‘ratio’ instead of ‘rate’. We changed our article as follows.

In the Introduction section on page 3, line 84-85:

“As of 22 March, 2021, the vaccine coverage ratio among eligible Korean population was only 1.57%, largely due to constraints on vaccine supply and delivery around the world at that time [4].”

2. *L81: I am confused about the 1.19%. This cannot be the percentage in the hospital where 63.3% got vaccinated. Is the 1.19% a figure for all of Korea? Also, it is important to report accessibility of the vaccine in this context. Vaccine distribution was characterized by bottlenecks in February and March this year.*

**Response:** The number 1.19% was produced with vaccine eligible Korean population as denominator. We corrected the miscalculation and revised our article accordingly.

In the Introduction section on page 3, line 84-85:

“As of 22 March, 2021, the vaccine coverage ratio among eligible Korean population was only 1.57%, largely due to constraints on vaccine supply and delivery around the world at that time [4].”

3. *L83: What is the completion rate? Is it the fraction of vaccinated individuals that complete the vaccination schedule? Again this would not be a rate (it is a dimensionless quantity, rates have unit 1/time).*

**Response:** We appreciate your detailed comment. We changed it to ‘percentage of completion’.

4. *L125-129: the statistical methods should be described in more detail, particularly whether the Yates correction was used with the chi-square tests, or what routines were run. Was it proc glm in SAS or glm() in R? etc.*

**Response:** Generally, Yates correction is used only in 2 by 2 case. In this study, we performed Chi-square test or Fisher’s exact test for 2 by r case not only for 2 by 2 case. As a results, we did not consider Yates correction into account. However, we would like to inform you that we performed Chi-square test or Fisher’s exact test with precision. Furthermore, we used proc logstic and proc freq procedure of SAS and R to produce figure. We revised our article accordingly.

In the subsection ‘*Statistical analysis’* in Method section on page 5, line 131-137:

“Categorical variables are presented as frequencies and percentages and were compared using the Chi-square test or Fisher’s exact test. Logistic regression was performed to identify predictive factors. With variables exhibiting significance in univariate analysis, as well as those with clinical relevance, we performed multivariate analysis. The validity of the variables was confirmed using the statistical variable selection method. All statistical analyses were performed using the R software version 4.0.2 (R Development Core Team, Vienna, Austria) and SAS software version 9.4 (SAS Institute Inc., Cary, NC, USA). Two-sided p-values < 0.05 were considered statistically significant.”

*Trivial comments:
L138: study -> the study*

**Response:** thank you for pointing out our mistake. We corrected it.

*P12L6: sSARS -> SARS*

**Response:** thank you for pointing out our mistake. We corrected it.

**References:**

1.	Kessels R, Luyten J, Tubeuf S. Willingness to get vaccinated against Covid-19 and attitudes toward vaccination in general. Vaccine. 2021. doi:10.1016/j.vaccine.2021.05.069 PMID:34119349

2.	Karlsson LC, Soveri A, Lewandowsky S, Karlsson L, Karlsson H, Nolvi S, et al. Fearing the disease or the vaccine: The case of COVID-19. Pers Individ Dif. 2021;172: 110590. doi:10.1016/j.paid.2020.110590 PMID:33518869

3.	Larntz K. Small-sample comparisons of exact levels for chi-squared goodness-of-fit statistics. Journal of the American Statistical Association. 1978;73: 253-263

4.	In the COVID-19 vaccine race, we either win together or lose together. Available: <https://www.who.int/news/item/10-02-2021-in-the-covid-19-vaccine-race-we-either-win-together-or-lose-together>. Accessed 30th August 2021.
